# Hidden Signals in Serum Protein Electrophoresis: Artificial Intelligence from Laboratory Pattern Recognition to Clinical Validation

**DOI:** 10.3390/diagnostics16203397

**Published:** 2026-10-19

**Authors:** Tiziana Biagioli, Luisa Lanzilao, Francesca Romano, Sara Ciullini Mannurita, Valentina Becherucci, Enza Aiello, Alessandra Fanelli, Alessandra Celli, Anna Maria Grazia Gelli, Edda Russo

**Affiliations:** 1 General Laboratory, Azienda Ospedaliero-Universitaria Careggi, 50134 Florence, Italy; biagiolit@aou-careggi.toscana.it (T.B.); lanzilaol@aou-careggi.toscana.it (L.L.); ciullinimannuritas@aou-careggi.toscana.it (S.C.M.);; 2 Clinical Pathology Laboratory, Santa Maria Annunziata Hospital, 50134 Florence, Italy; valentina.becherucci@uslcentro.toscana.it; 3 Clinical Pathology Laboratory Unit, S. Giuseppe Hospital, Azienda USL Toscana Centro, 50053 Empoli, Italy; enza.aiello@unifi.it (E.A.); annamariagrazia.gelli@uslcentro.toscana.it (A.M.G.G.); 4 Clinical Pathology Laboratory Unit, San Jacopo Hospital, Azienda USL Toscana Centro, 51100 Pistoia, Italy; alessandra1celli@ulscentro.toscana.it

**Keywords:** artificial intelligence, serum protein electrophoresis, monoclonal gammopathy, M-protein, machine learning, high-dimensional signal analysis, clinical validation, laboratory medicine

## Abstract

Serum protein electrophoresis (SPE) remains a central laboratory method for the detection and characterization of monoclonal gammopathies and other dysproteinemic patterns. Despite substantial analytical automation, interpretation still relies largely on graphical representation and expert visual assessment, making low-amplitude or borderline abnormalities particularly challenging. Artificial intelligence (AI), including machine-learning (ML) and deep-learning (DL) approaches, has increasingly been applied to SPE, with high performance reported for automated recognition of established abnormalities, particularly M-proteins, i.e., monoclonal immunoglobulins or immunoglobulin fragments produced by clonal plasma cells. However, most models have been developed against predefined diagnostic targets and evaluated using expert interpretation, immunofixation, or immunotyping as reference standards. Improved recognition of known abnormalities should therefore not be equated with discovery of additional clinically meaningful information. Modern electrophoretic platforms generate machine-readable numerical signals that may preserve features not explicitly considered during routine graphical interpretation. This creates the possibility of extending AI-assisted SPE from conventional pattern recognition toward detection of low-amplitude, distributed, or sub-visual signal characteristics. Yet current literature has focused predominantly on algorithmic performance, while the validation pathway required to determine whether additional AI-detected features are biologically meaningful and clinically actionable remains insufficiently defined. Evidence is strongest for automated M-protein recognition, including low-amplitude abnormalities, whereas non-canonical systemic patterns, longitudinal signal analysis, and AI-assisted decomposition of therapeutic monoclonal antibody interference remain exploratory. This critical review addresses this translational gap by evaluating AI applications according to the nature of detected information, strength of supporting evidence, and proximity to clinical implementation. We propose a translational framework addressing four complementary domains: analytical validity, biological validity, clinical validity, and clinical utility. The evidence required in each domain depends on the intended claim and clinical use.

## 1. Introduction

Serum protein electrophoresis (SPE) remains a core method in laboratory medicine for the detection and characterization of dysproteinemias, particularly monoclonal gammopathies, while also providing information on broader inflammatory, immune-mediated, hepatic, and neoplastic processes [1,2,3,4]. Despite substantial automation of the analytical phase, interpretation still relies largely on graphical representation of the electrophoretic profile and expert visual assessment. This approach is generally effective for well-defined abnormalities but becomes less robust when monoclonal components are small, poorly demarcated, or embedded within heterogeneous polyclonal backgrounds, where subtle morphological changes may be inconsistently recognized [1,2,3,4].

Artificial intelligence (AI) has emerged as a potential means of reducing this interpretative variability. Machine-learning (ML) and deep-learning (DL) approaches have been applied to electrophoretic profiles, extracted features, and numerical SPE data, with several large retrospective studies reporting high performance for automated recognition of M-proteins and other predefined electrophoretic abnormalities [5,6,7,8,9,10,11,12]. Collectively, these studies establish an increasingly convincing role for AI in automating and extending conventional pattern recognition, including the detection of low-concentration monoclonal components that may be difficult to identify visually.

However, this progress also defines the current boundary of the field. Most available models have been developed using diagnostic categories whose clinical meaning is already established and have been evaluated against expert interpretation, immunofixation, immunotyping, or related laboratory reference procedures [5,7,8,9,10]. These studies support the feasibility of automated pattern recognition, including detection of low-concentration monoclonal components, with performance varying by task and validation setting. This distinction becomes particularly relevant when SPE is considered not only as a graphical pattern, but as a machine-readable quantitative signal. Modern electrophoretic platforms generate sequential numerical measurements that can be analysed computationally before, or independently of, their conventional graphical representation [6,7,11]. Such data potentially preserve low-amplitude, distributed, and local signal characteristics that are not explicitly considered during routine visual interpretation. AI may therefore expand the portion of the electrophoretic signal that is computationally accessible. Yet greater access to signal complexity does not necessarily translate into greater diagnostic information. A computationally detectable feature may reflect disease biology, but it may equally represent physiological heterogeneity, transient inflammation, pre-analytical variation, analytical noise, preprocessing effects, or platform-specific characteristics. Modern electrophoretic systems generate sampled numerical signals through gel densitometry or capillary detection. The graphical electrophoretic profile is a visual representation of that signal. Computational analysis can evaluate characteristics that may not be explicitly assessed during routine visual interpretation, including local shape and relationships between regions [6,7,11]. Exported numerical data may already have undergone instrument-specific processing and should not automatically be described as raw signals. The information retained depends on acquisition, preprocessing, and export format; numerical representation alone does not establish additional biological or clinical information.

This represents the central translational gap in the current AI-SPE literature. Previous research has predominantly addressed whether algorithms can recognize predefined electrophoretic abnormalities accurately, whereas considerably less attention has been given to the validation pathway required to determine whether additional AI-detected features are analytically valid, biologically meaningful, clinically valid, and ultimately useful for patient management. This issue becomes particularly important as AI applications move toward low-amplitude abnormalities, non-canonical electrophoretic patterns, longitudinal signal analysis, and other features lying beyond conventional visual classification.

Accordingly, this review does not primarily examine which AI architecture performs best in SPE. Instead, it critically evaluates what type of electrophoretic information current AI approaches actually detect, how strong the supporting evidence is, and what evidence is still required before additional signal features can be considered clinically actionable.

We distinguish established applications from emerging and conceptual uses and propose a translational framework addressing four complementary domains: analytical validity, biological validity, clinical validity, and clinical utility.

## 2. Literature Search and Critical Appraisal

A structured narrative literature search was conducted to identify studies investigating the application of artificial intelligence (AI), machine learning (ML), and deep learning (DL) to serum protein electrophoresis (SPE). PubMed, Scopus, and Web of Science were searched for English-language publications addressing serum protein electrophoresis, capillary electrophoresis, artificial intelligence, machine learning, deep learning, M-protein detection, monoclonal gammopathy, and automated interpretation. The search was performed in August 2026. Additional relevant publications were identified through reference-list screening and citation tracking of key studies and methodological reviews.

During manuscript revision in October 2026, targeted searches of PubMed and publisher records were used to verify cited studies and identify additional methodological references on biological variability and incremental predictive value. These targeted checks did not constitute a repeat search of all three databases.

Particular attention was given to original studies evaluating automated SPE interpretation, M-protein detection, low-amplitude electrophoretic abnormalities, and applications based on numerical or high-dimensional signal representations. Studies addressing therapeutic monoclonal antibody interference were also considered where relevant to the conceptual discussion of signal separation and computational deconvolution.

Studies were considered relevant when they evaluated computational methods applied directly or indirectly to SPE data and reported their analytical or clinical target, data representation, validation strategy, and performance. Methodological reviews and established laboratory literature were included to provide analytical and clinical context. Studies were not excluded solely on the basis of model architecture or reported performance; rather, their contribution was considered in relation to the maturity of the intended clinical application.

Given the heterogeneity of study designs, analytical platforms, AI methodologies, reference standards, and reported outcomes, no meta-analysis was performed. Instead, representative studies were critically appraised according to the strength and translational maturity of the supporting evidence. The assessment considered dataset size and representativeness, definition of the intended clinical target, quality of the reference standard, analytical platform and input representation, independence of development and validation cohorts, external validation, potential for overfitting, and proximity to prospective clinical implementation. Particular attention was given to whether reported performance reflected improved recognition of a predefined electrophoretic abnormality or evidence that additional AI-detected signal features provided information beyond conventional interpretation.

The qualitative appraisal was informed by principles derived from TRIPOD + AI and PROBAST + AI [13,14]. Because this was a structured narrative review and reporting was heterogeneous across studies, no numerical risk-of-bias score or formal overall quality classification was assigned. Evidence was instead considered along a translational continuum ranging from proof-of-concept and internal validation to external or multicenter validation, prospective workflow evaluation, and routine clinical implementation. Translational maturity was therefore assessed according to the robustness and clinical relevance of the evidence rather than diagnostic performance alone. Full texts were consulted where accessible; otherwise, appraisal was restricted to information available in abstracts and publisher records. Unavailable methodological details were not treated as demonstrated study limitations.

## 3. From Electrophoretic Profiles to Machine-Readable Signals: Analytical Foundations and Current Evidence

The application of AI to SPE depends not only on model architecture but also on how electrophoretic information is represented. SPE data can be provided to computational models as rendered curves or images, conventional fraction measurements, engineered features, or numerical sequences derived directly from the electrophoretic signal. These representations are not equivalent: each preserves different aspects of the analytical output and therefore determines which features a model can access and learn [5,6,7,8].

From a computational perspective, different AI architectures interact with these representations in distinct ways. Convolutional neural networks (CNNs) are particularly effective when electrophoretic information is represented as curves or images, allowing hierarchical extraction of local morphological features without predefined descriptors. Conversely, numerical electrophoretic sequences can be analysed directly by one-dimensional CNNs (1D-CNNs), transformer-based architectures, or conventional supervised machine-learning algorithms. Random forests and support vector machines can model relationships among input variables, but do not inherently encode electrophoretic ordering or local neighbourhood structure. Whether architectures that explicitly represent these properties improve performance requires task-specific comparison. Early neural-network systems, including CASPER and Neurosoft-Sebia, evaluated automated recognition of abnormal electrophoretic profiles [15,16].

Most AI applications developed to date have treated SPE primarily as a pattern-recognition problem. Deep-learning models can learn morphological characteristics directly from electrophoretic profiles, while conventional machine-learning approaches can operate on extracted features or numerical representations. Large studies such as SPECTR and AIRSPE have demonstrated high performance in recognizing established abnormalities, including M-spikes and low-concentration monoclonal components [5,10]. Similarly, numerical capillary electrophoresis data have been successfully analysed using ensemble and feature-based ML approaches for M-protein detection [7,8]. Collectively, this evidence establishes that clinically relevant classification does not require exclusive reliance on the graphical representation traditionally inspected by laboratory professionals.

The implications of numerical signal analysis, however, extend beyond replacing visual interpretation with automated classification. Wang et al. showed that feature representation itself influences model performance and demonstrated expert-level M-protein classification and localization using a U-Net–Transformer architecture [11]. This observation highlights a broader methodological principle: the optimal representation depends on the clinical question being asked. Detecting a predefined M-spike and searching for subtle distributed changes within an otherwise non-canonical profile are fundamentally different tasks and may require different representations of the same underlying electrophoretic information.

This distinction becomes particularly important when AI is proposed as a means of accessing information beyond established diagnostic categories. Models trained on conventional labels may achieve excellent discrimination while remaining constrained by those same labels. More granular numerical representations may preserve local relationships, low-amplitude variations, or distributed signal characteristics that may not be explicitly assessed during visual interpretation. Yet a more detailed representation does not establish biological relevance. A computationally detectable feature may reflect disease-related information, physiological variability, or analytical structure. Detecting additional features is only the first step; their reproducibility and clinical relevance must be demonstrated separately. Preprocessing further complicates this transition. Electrophoretic signals are influenced by analytical platform, signal acquisition, baseline behaviour, migration alignment, instrument-specific characteristics, detector ageing, reagent lot differences, and other technical sources of variation. Baseline correction, normalization, noise management, and profile alignment can improve comparability, but may also suppress, amplify, or reshape precisely the low-amplitude features that computational models are intended to detect. Consequently, preprocessing, input standardization, and cross-platform evaluation should be regarded as integral components of analytical validation rather than preliminary technical procedures [8,9]. In multicenter applications, insufficient control of these effects creates the risk that models learn laboratory- or platform-specific signatures rather than transferable biological patterns. Lahtiharju et al. applied the SPECTR methodology to a Finnish cohort with local model training [9], whereas Cheung et al. evaluated models developed at one hospital using data from another hospital [12]. These designs address different aspects of transferability and should be distinguished from cross-platform validation.

Finally, conventional performance metrics address discrimination against a predefined reference standard, not the biological or clinical significance of the detected information. Current high-performing SPE models have predominantly been evaluated against immunofixation, immunotyping, or expert-defined electrophoretic categories [5,7,8,9,10,11]. High sensitivity, specificity, accuracy, or area under the receiver operating characteristic curve therefore demonstrates successful recognition of a known target but cannot, by itself, establish the existence of a clinically meaningful sub-visual signature. Once AI moves beyond automated recognition of established abnormalities, the principal problem is no longer classification performance but validation of the signal itself.

## 4. Beyond Pattern Recognition: What Does the Evidence Support?

The clinical relevance of AI in SPE depends primarily on what the model detects and how convincingly that information has been validated, rather than on the complexity of the algorithm. Current applications occupy markedly different positions along this continuum. At one end, AI-assisted recognition of established electrophoretic abnormalities, particularly monoclonal proteins, is supported by large retrospective datasets and, in selected studies, independent validation [5,7,8,9,10,11]. At the other end, proposed applications involving non-canonical systemic patterns, longitudinal sub-visual changes, or computational separation of overlapping therapeutic and endogenous monoclonal components remain exploratory or conceptual.

**Figure 1:**
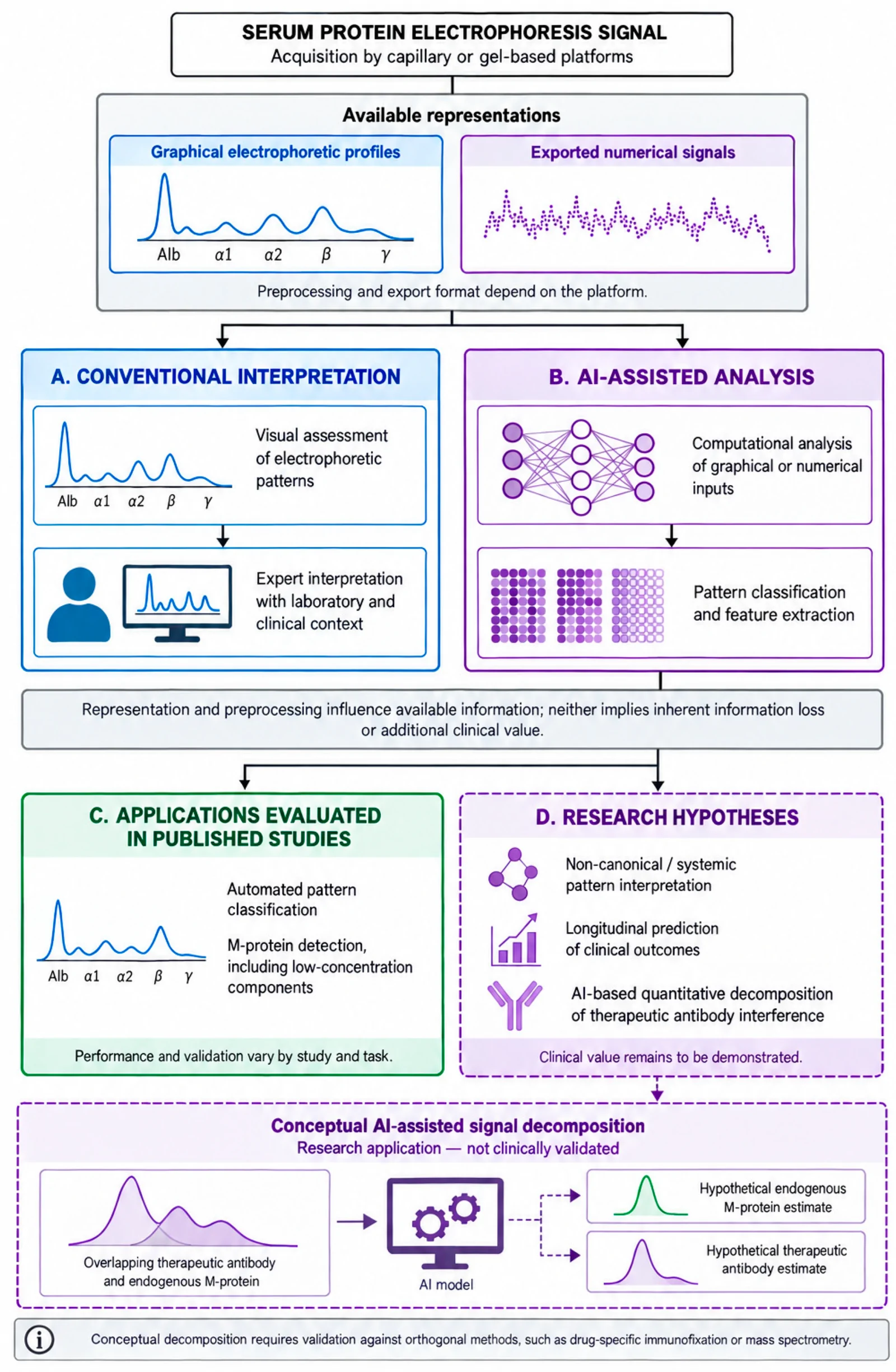
Conventional interpretation and AI-assisted analysis of serum protein electrophoresis: evaluated applications and research hypotheses. Graphical profiles and exported numerical signals provide representations of the electrophoretic signal whose information content depends on platform-specific acquisition, preprocessing, and export procedures. Conventional interpretation relies on visual assessment and laboratory and clinical context (**A**), whereas AI-assisted analysis can use graphical or numerical inputs for pattern classification and feature extraction (**B**). Applications evaluated in published studies include automated pattern classification and M-protein detection, with performance and validation varying by study and task ((**C**), solid green outline). Interpretation of non-canonical or systemic patterns, longitudinal prediction of clinical outcomes, and AI-based quantitative decomposition of therapeutic antibody interference are presented as research hypotheses ((**D**), dashed purple outline). The lower inset illustrates a conceptual separation of overlapping therapeutic antibody and endogenous M-protein signals; the depicted outputs are hypothetical estimates, not validated measurements. Such decomposition requires validation against orthogonal methods, including drug-specific immunofixation or mass spectrometry. Curves are schematic. AI, artificial intelligence; SPE, serum protein electrophoresis.

This distinction is essential because three different claims are often conflated: improved recognition of a known abnormality, detection of additional signal features, and discovery of clinically meaningful information. The first can be established against existing laboratory reference procedures. The second requires evidence that the detected feature is analytically reproducible. The third requires substantially stronger biological and clinical validation. The available SPE literature predominantly supports the first claim, provides emerging evidence for the second, and remains limited for the third. The following applications are therefore considered according to this hierarchy of evidence rather than according to model architecture or reported diagnostic performance alone. Figure 1 summarizes this transition from conventional visual interpretation to AI-driven signal analysis and places the main established, emerging, and conceptual applications within a single laboratory-oriented framework.

### 4.1. Established and Emerging Evidence: From M-Protein Recognition to Low-Amplitude Abnormalities

Recognition of monoclonal proteins represents the most mature application of AI-assisted SPE. Multiple studies using deep learning, feature-based machine learning, and numerical electrophoretic data have demonstrated high performance for M-protein detection [5,7,8,9,10,11,12]. Importantly, the clinical target in these studies is already biologically and diagnostically defined, allowing algorithmic predictions to be compared with expert interpretation, immunofixation, immunotyping, or other established laboratory procedures. AI therefore operates within a diagnostic framework in which the abnormality of interest and its clinical meaning are already known.

Within this established application, low-amplitude monoclonal abnormalities represent a particularly relevant extension of conventional pattern recognition. Small M-proteins may be difficult to distinguish from polyclonal background when their concentration approaches the analytical or interpretative limits of routine SPE. AI-assisted analysis can improve recognition at this boundary, and recent evidence, particularly from AIRSPE, supports increased sensitivity for low-concentration M-spikes [10]. This represents a clinically plausible advantage because equivocal AI findings can still be evaluated against independent confirmatory methods.

The interpretation of this improvement nevertheless requires precision. A monoclonal component that escapes routine visual recognition but is subsequently confirmed by immunofixation is sub-visual in an operational sense, but not biologically novel. AI has improved access to an established diagnostic signal; it has not demonstrated the existence of a previously unknown disease signature. This distinction matters because increasingly sensitive computational detection is sometimes implicitly interpreted as evidence of earlier disease recognition. Current studies do not establish that an AI-detected electrophoretic alteration precedes conventional biochemical or clinical evidence of monoclonal gammopathy, nor that detecting it earlier improves patient outcomes.

The next evidentiary step should therefore move beyond retrospective agreement with established reference procedures. Prospective studies should specifically evaluate AI-positive but conventionally equivocal profiles, determining whether they predict subsequent confirmation of monoclonality, clinically relevant progression, organ involvement, or changes in patient management beyond existing diagnostic pathways. Only such evidence can establish whether increased computational sensitivity provides incremental clinical value rather than simply increasing the number of abnormalities referred for confirmatory testing.

### 4.2. Beyond Monoclonal Gammopathies: Demonstrated Pattern Recognition and Hypothesized Systemic Signatures

Inflammatory, autoimmune, infectious, hepatic, and neoplastic conditions can be associated with changes across albumin and globulin fractions. Conventional SPE interpretation describes some of these changes using established categories, including polyclonal hypergammaglobulinemia and beta–gamma bridging [1,2,3]. These patterns are not necessarily specific to a single disease. Here, “non-canonical” refers to proposed computational features that do not correspond to established electrophoretic categories, rather than to all patterns occurring outside monoclonal gammopathies.

Published AI studies have demonstrated recognition of some conventional non-M-spike patterns. Chabrun et al., for example, evaluated SPECTR against expert-defined categories that included restricted immunoglobulin heterogeneity and beta–gamma bridging [5]. This supports automated recognition of predefined electrophoretic morphology. It does not establish that the model identified the underlying cause of these patterns or discovered a previously unrecognized disease-specific signature. A separate hypothesis is that numerical SPE profiles contain distributed features associated with systemic disease that are not adequately captured by conventional fractions or visual categories. Candidate features might include local changes in signal shape or relationships between electrophoretic regions. Their evaluation is proposed here as a research direction, not presented as an experimentally established application. The evidence reviewed does not establish that such features distinguish specific systemic or autoimmune diseases, predict their evolution, or add clinically useful information beyond routine laboratory measurements. Nor can a particular model architecture be assumed to provide these capabilities without a direct comparison for the intended task.

Testing this hypothesis requires a reference assessment matched to the claim. A model intended to reproduce an electrophoretic category can be evaluated against expert interpretation. A model intended to identify a systemic disease requires an independently established clinical diagnosis or outcome definition; an electrophoretic label alone is insufficient. Disease activity, treatment, organ dysfunction, and inflammatory burden must also be considered, because a model may distinguish patient groups using features associated with these factors rather than with the disease itself. A useful study would therefore compare patients with the target condition against clinically relevant alternative diagnoses, including conditions with similar inflammatory or protein changes, rather than only healthy controls. Candidate features would require analytical reproducibility and evaluation in independent populations. Claims of incremental value would additionally require comparison with conventional SPE fractions and routinely available clinical and laboratory variables. Orthogonal protein measurements could help investigate biological attribution, while longitudinal sampling would be necessary to test claims of change preceding a clinical event.

Accordingly, automated recognition of established non-M-spike patterns and discovery of new systemic signatures should be treated as different applications. The former has supporting evidence for selected categories; the latter remains a hypothesis requiring validation for a specified clinical use.

### 4.3. Therapeutic Monoclonal Antibody Interference: Established Methods and Computational Research Directions

Therapeutic monoclonal antibodies (t-mAbs) can generate bands or peaks on SPE and immunofixation electrophoresis (IFE), complicating the distinction between treatment-related signals and endogenous M-proteins [17,18,19]. Conventional IFE identifies immunoglobulin isotype but may not resolve the origin of a band when the drug and endogenous protein share the same isotype and migration position. Treatment history and pretreatment profiles can support interpretation, but do not invariably resolve co-migration. Experimental characterization of therapeutic antibody migration, including the study by Milandri et al. [20], provides information for recognizing possible interference rather than proof that overlapping components can be computationally separated.

Drug-specific IFE assays address this problem by introducing an additional source of analytical specificity. DIRA and the daratumumab-specific Hydrashift assay use an anti-daratumumab reagent to alter the drug’s migration, allowing assessment of a residual endogenous band [18,19,21]. McCudden et al. demonstrated discrimination between daratumumab and residual M-protein in spiked and patient samples, together with reproducibility across days, operators, and assays [19]. Kirchhoff et al. reported 280 Hydrashift assays in 96 patients, including 43 with co-migrating daratumumab/IgGκ bands [21]. These methods have direct evidence for resolving daratumumab interference on IFE, but their specificity is restricted to the targeted drug. Resolution on IFE should also be distinguished from correction of the M-protein concentration measured on the original SPE trace.

Mass spectrometry uses molecular mass or peptide identity to distinguish therapeutic antibodies from endogenous immunoglobulins. Kohlhagen et al. evaluated Mass-Fix and the higher-resolution miRAMM method using sera from 86 patients with IgGκ M-proteins spiked with therapeutic antibodies [22]. Mass-Fix resolved the interference in 87% of spiked samples; miRAMM resolved the remaining cases. All 21 samples from patients receiving the evaluated drugs were resolved by Mass-Fix. These results demonstrate that separation depends on the analytical method and should not be assumed for every mass-spectrometric platform. In a proof-of-principle study involving three patients receiving daratumumab and nivolumab, Noori et al. evaluated double Hydrashift and clonotypic peptide-based mass spectrometry, illustrating approaches to interference from combined antibody treatments [23].

Computational analysis already contributes to automated mass-spectrometric workflows. Barnidge et al. described peak-identification and modeling algorithms within the EXENT system and evaluated 109 samples from 34 patients receiving daratumumab-containing treatment [24]. Daratumumab was detected in 103 samples (94.5%). This detection rate should not be interpreted as a universal rate of successful separation from endogenous M-protein. The authors identified limitations when drug and endogenous light-chain peaks were close in mass or differed substantially in abundance, and when pretreatment information was unavailable. This is evidence for computational interpretation of mass spectra, not for AI-based decomposition of SPE profiles.

For AI-SPE, identifying a profile that warrants investigation for drug interference and estimating the separate concentrations of co-migrating components are different tasks. The latter remains a research hypothesis in the evidence reviewed here. A reproducible drug migration pattern could inform a model, but migration position alone does not establish molecular identity. When two components contribute indistinguishable signals, decomposition may depend on assumptions that cannot be verified from the SPE trace alone. Increasing model complexity does not, by itself, resolve this ambiguity.

A validation study should therefore define whether the intended output is an interference flag, attribution of a band, or quantitative separation. Controlled mixtures with known drug and endogenous M-protein concentrations would test analytical recovery, while paired pretreatment and on-treatment samples would assess performance in clinical specimens. Drug-specific IFE could support qualitative attribution; quantitative claims would require an independently validated quantitative comparator. Evaluation should include co-migrating IgGκ components, low-concentration residual proteins, different drug-to-M-protein ratios, and independent analytical settings. Until such evidence is available, AI-based interference flags should prompt appropriate confirmatory testing rather than justify removal of a suspected drug contribution from the reported M-protein.

### 4.4. From Algorithmic Performance to Translational Readiness

Reported performance should be interpreted in relation to the dataset, reference assessment, and validation design rather than model architecture alone (Table 1). The studies address partly different tasks, including recognition of expert-defined abnormalities, detection of immunofixation- or immunotyping-confirmed M-proteins, and peak localization or quantification. Their performance estimates are therefore not directly interchangeable. In particular, agreement with expert interpretation measures reproduction of the reported classification, whereas comparison with an independent confirmatory assay addresses a different diagnostic question.

Validation strategies also differ. SPECTR was evaluated in an independent external cohort of 70,362 profiles [5], and Cheung et al. tested models developed at one hospital using data from a second hospital [12]. By contrast, Lahtiharju et al. trained models locally using the SPECTR methodology [9]. This provides evidence for application of the methodology in another setting, but should be distinguished from external validation of an unchanged pretrained model. Likewise, a held-out dataset from the development environment does not establish cross-center or cross-platform transportability.

Direct comparisons within individual studies illustrate why overall claims of superiority require qualification. In Elfert et al., the random forest classifier achieved higher specificity than the expert group (99.8% versus 88.9%) but lower sensitivity (89.9% versus 94.3%) [8]. AIRSPE showed the opposite trade-off in its internal test set: sensitivity exceeded manual interpretation (approximately 95% versus 69%), whereas specificity was lower (86.97% versus 96.52%) [10]. Its external-test sensitivity was 89.58%, with specificity of 87.18%. These findings support different operating characteristics rather than uniformly better performance across all diagnostic objectives.

Dataset composition can affect both model development and estimates of diagnostic performance. Cohorts assembled from specialist hematology services, patients undergoing follow-up for known monoclonal gammopathies, or samples selected for immunofixation may differ from unselected routine SPE requests. For example, the AIRSPE test cohorts required available immunofixation results [10]. This strengthens reference verification within the evaluated samples but restricts the population represented. Conversely, when confirmatory testing is performed only for suspicious profiles, apparently negative samples may contain unrecognized abnormalities. Reference-label errors and selective verification should therefore be considered separately from algorithmic errors.

Class imbalance requires interpretation in relation to the intended population. Elfert et al. included 4273 immunotyping-positive and 65,449 negative samples [8]. An excess of negative samples may reflect routine practice rather than defective sampling, but overall accuracy can obscure inadequate detection of the minority class. Evaluation should report class-specific performance and confidence intervals, together with predictive values at the observed prevalence. Precision–recall analysis can complement ROC analysis, although it is also prevalence-dependent. Class weighting or resampling should be justified rather than applied automatically; if used, these procedures should remain within the training process, and their effects on probability calibration should be assessed in representative evaluation data [13,14].

Spectrum bias is a separate concern. Performance estimated from clearly defined M-spikes and uncomplicated negative profiles may not represent performance for low-concentration components, beta-region abnormalities, oligoclonal patterns, or treated patients with potentially interfering therapeutic antibodies. Sensitivity and specificity can change when the distribution of these presentations differs, even without a change in the algorithm. Studies should therefore describe the clinical and electrophoretic spectrum and report performance in relevant subgroups where sample size permits, rather than relying exclusively on pooled estimates.

Patient-level dependence also matters. Sopasakis et al. analysed 67,073 samples from 32,490 patients [7], illustrating why profile counts should not be equated with independent patient counts. If serial samples from the same patient enter both development and evaluation sets, persistent patient-specific patterns may produce optimistic performance estimates. This is a potential risk to assess, not evidence that leakage occurred in a particular study. Data partitions should match the intended use, with patient-level separation for evaluation in previously unseen patients. Learned preprocessing, feature selection, and threshold optimization should likewise exclude the final test data [13,14].

Differences in referral pathways, age distribution, comorbidities, treatment exposure, and the balance between initial investigation and disease monitoring may further limit transportability. These population differences may coincide with platform or laboratory differences, making their effects difficult to disentangle. External evaluation should characterize both the clinical population and the analytical setting, report subgroup performance and calibration where applicable, and distinguish evaluation of the original model from performance after local updating. A change in prevalence alone can alter predictive values; accompanying changes in case mix can also alter discrimination and threshold-specific performance. Applicability should therefore be judged against the intended deployment population rather than inferred from geographic diversity or dataset size alone [13,14]. A further distinction concerns what is being validated. For established targets such as M-proteins, external validation assesses whether model performance is transferable to the intended population and analytical setting. For additional or sub-visual signal features, reproducibility across independent datasets does not by itself establish clinical relevance. Clinical claims require evidence linking the feature to a defined clinical state or outcome, while claims of added value require comparison with existing diagnostic pathways. Biological attribution supports interpretation, but when it remains unresolved, claims about the feature’s origin or disease specificity should remain limited.

Taken together, the current literature supports a progressive evidence hierarchy. Automated recognition of established electrophoretic abnormalities has reached internal and, for selected models, independent retrospective validation [5,7,8,9,10,11,12]. Evidence becomes substantially less mature as the intended use moves toward additional signal detection, biological attribution, longitudinal prediction, or clinically actionable decision support. Prospective workflow studies and outcome-based evaluations remain comparatively limited. Validation should match the intended claim: broader deployment requires evidence of transportability, whereas claims of added clinical information require comparison with existing laboratory and clinical assessment.

This distinction provides the rationale for a validation framework specifically addressing the transition from computational signal detection to clinically meaningful laboratory information.

## 5. From Signal Detection to Clinical Meaning: A Validation Framework

As AI increases the amount of electrophoretic information accessible to computational analysis, the central challenge shifts from detecting abnormalities to determining which detected features represent clinically meaningful information. This distinction is particularly relevant for low-amplitude, distributed, or non-canonical signal changes that do not correspond directly to established electrophoretic categories. Such features may reflect early or previously unrecognized biological processes, but they may equally arise from physiological variation, pre-analytical conditions, analytical noise, preprocessing, or platform-specific effects. Computational detectability should therefore be regarded as the beginning of validation rather than evidence of biomarker status. We propose four complementary domains for evaluating additional AI-detected SPE features: analytical validity, biological validity, clinical validity, and clinical utility. Within this framework, analytical validity concerns the reliability of the measured feature and model output; biological validity concerns evidence supporting the proposed biological interpretation; clinical validity concerns performance for a defined clinical target; and clinical utility concerns the consequences of using the information in practice. These domains guide evidence development rather than impose a fixed experimental sequence. In particular, incomplete mechanistic understanding does not preclude demonstration of clinical validity, but limits claims about the biological origin or disease specificity of a feature.

For each application, the intended population, analytical platform, model output, and decision to be supported should be specified before validation. Primary endpoints and acceptance criteria should be prespecified and justified according to the intended use and the consequences of error. Table 2 provides proposed operational criteria for each domain; these are not universal numerical thresholds or a formal grading system.

### 5.1. Analytical Validity

Analytical validation should characterize measurement reliability and assess whether an AI-detected feature is sensitive to technical artefacts.

This includes assessment of within-run and between-run reproducibility, sample stability, preprocessing effects, migration alignment, instrument variability, and robustness across analytical platforms and laboratories. Particular attention is required for low-amplitude features because baseline correction, smoothing, normalization, and noise reduction may modify precisely the signal characteristics that computational models are intended to exploit.

Importantly, analytical reproducibility should be demonstrated not only for model predictions but also for the underlying computational features themselves. A feature that appears consistently informative within one analytical environment may disappear after changes in preprocessing, platform, or signal acquisition. Consequently, validation should address both algorithmic reproducibility and feature reproducibility to support interpretation of the detected signal.

**Figure 2:**
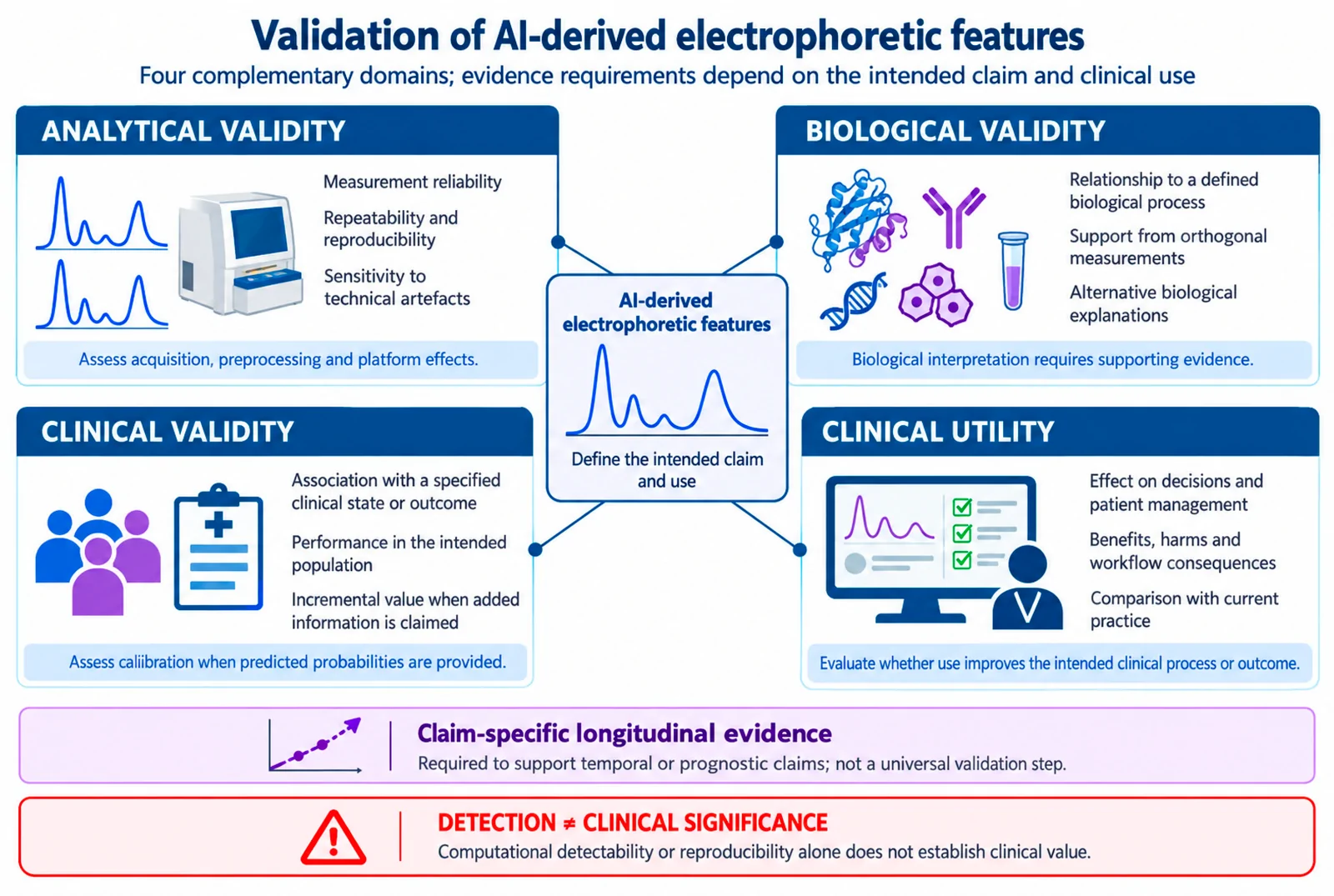
Translational framework from AI-detected electrophoretic signals to clinically actionable information. Computational detection alone does not establish biomarker status. The framework distinguishes analytical validity, biological validity, clinical validity, and clinical utility. These assessments address complementary questions; insufficient evidence in one domain limits the corresponding claim, while incomplete biological attribution does not necessarily preclude demonstration of clinical validity.

The scope of analytical validation should match the intended deployment. A model developed for one platform may initially be validated within that environment, but its performance cannot be assumed to transfer to other systems. Multicenter and cross-platform evaluation is required to support broader claims of transportability. Figure 2 summarizes the four complementary validation domains and the role of longitudinal evidence in supporting temporal or prognostic claims.

### 5.2. Biological Validity

Biological validity concerns whether the detected feature reflects a defined biological process.

This assessment is particularly challenging for AI-derived SPE features because electrophoretic profiles integrate multiple protein populations and may change in response to inflammation, immune activation, hepatic function, nutritional status, treatment, and other physiological or pathological conditions.

Biological validation should therefore seek independent evidence linking the computational feature to its proposed biological origin. Depending on the application, this may involve immunofixation, immunotyping, quantitative immunoglobulins, serum free light chains, mass spectrometry, proteomic characterization, inflammatory biomarkers, or other orthogonal laboratory measurements. The objective is not simply to demonstrate statistical association with a diagnostic label, but to determine what biological phenomenon the detected signal actually represents.

This distinction is essential for proposed non-canonical or systemic SPE signatures. A model may distinguish two patient groups with high accuracy without identifying a disease-specific biological feature. Correlation with diagnosis alone should therefore not be considered evidence of biological validity. Related methodological challenges have been discussed for AI applications in non-blood biological fluids [25]. This provides context for the validation framework, rather than experimental evidence for AI-SPE.

### 5.3. Clinical Validity

Clinical validity concerns the ability of the feature or model to identify or predict a specified clinical state or outcome in the intended population.

Importantly, clinical validity should not be reduced to discrimination between predefined case and control groups. Claims of added diagnostic or prognostic information require demonstration of incremental value beyond existing laboratory and clinical variables. Depending on the intended use, relevant endpoints may include confirmation of monoclonality, disease progression, development of organ involvement, treatment response, or another clinically meaningful outcome. Performance across clinically relevant subgroups should be evaluated, together with calibration when the model provides predicted probabilities, rather than relying exclusively on global discrimination metrics.

Longitudinal evidence may be particularly informative at this stage. If a putative sub-visual feature is proposed as an early disease signal, its value should be demonstrated by showing that its presence or evolution precedes and predicts a clinically relevant event rather than merely correlating with established disease at a single time point.

### 5.4. Clinical Utility

Clinical utility concerns the consequences of using AI-derived information within a defined laboratory or clinical pathway. An analytically reproducible feature or an accurate classification may offer little practical benefit if it does not improve decisions about confirmatory testing, follow-up, or patient management. Evaluation should therefore compare AI-assisted interpretation with current practice, accounting for both additional clinically relevant findings and the investigations generated to identify them.

False-positive classifications can increase requests for immunofixation or other confirmatory assays. In the AIRSPE internal test cohort, 907 of 6963 IFE-negative samples were classified as positive by AI, compared with 242 by manual interpretation [10]. These are false-positive classifications relative to IFE, not observed counts of unnecessary tests: all samples in that cohort already had IFE results. The findings illustrate a potential workload consequence of the sensitivity–specificity trade-off, but do not establish its magnitude in routine deployment. False-positive findings should also be distinguished from confirmed abnormalities whose additional detection has uncertain clinical benefit. An AI-positive result that is not confirmed may require review of the trace, sample quality, treatment history, and reference-method limitations. A confirmed small monoclonal component presents a different question: whether detecting it changes management or outcomes. Neither discordance nor low concentration alone establishes that an investigation is unnecessary. Conversely, increased detection should not be interpreted as clinical benefit without assessing its downstream consequences.

Potential harms include repeated sampling, avoidable specialist referrals, prolonged surveillance of unresolved findings, and patient anxiety if an unconfirmed alert is communicated as a diagnosis. In patients receiving therapeutic monoclonal antibodies, incorrect attribution of a drug-related band to residual endogenous M-protein could also affect response assessment [17,18,19,21]. These are risks to evaluate prospectively, rather than outcomes demonstrated by retrospective classification studies. Reports should distinguish a computational alert, an electrophoretic finding, and a confirmed laboratory interpretation.

The effect on workload cannot be inferred from algorithm processing time alone. Automated review may save time for straightforward profiles while increasing the time required to resolve discordant or indeterminate results. We therefore propose measuring the proportion of samples referred for manual review, staff time per sample, confirmatory assays per 1000 SPE requests, repeat investigations, and turnaround time. These measures should be assessed alongside missed clinically relevant abnormalities, the yield of additional testing, and changes in patient management. Where identifiable, additional tests and staff time per additional clinically relevant finding would help quantify the practical trade-off. Implementation studies should prespecify the action associated with each AI output, criteria for confirmatory testing, and the handling of unresolved results. Decision thresholds should reflect the intended role of the model and the relative consequences of missed abnormalities and additional investigations. For models providing clinically interpretable probabilities, decision-curve analysis may help assess net benefit across relevant thresholds [13,26], but it does not replace observation of actual testing, workload, and patient outcomes. Evidence of clinical utility should remain limited to the benefits demonstrated: faster reporting or fewer manual reviews does not by itself establish better patient care.

## 6. Research Priorities: Longitudinal and Multimodal SPE

The next phase of AI-assisted SPE research should focus less on increasing model complexity and more on generating the evidence required to determine whether additional electrophoretic information has clinical value. Two directions appear particularly relevant: longitudinal signal analysis and multimodal integration. Both may extend SPE beyond cross-sectional pattern recognition, but neither should be considered clinically established.

Longitudinal AI-SPE analysis could evaluate changes relative to an individual baseline, but such changes must be distinguished from pre-analytical variation, analytical imprecision, and within-person biological variability. A single previous profile is insufficient to characterize an individual’s expected fluctuation. We propose establishing baseline variability from repeated samples collected during a clinically characterized period, with the number and spacing of measurements determined by the intended application. Stability should not be defined solely by the absence of change in the feature under investigation, as this would underestimate its variability.

Serial sampling protocols should specify collection intervals and record actual sampling dates and timing relative to treatment, particularly therapeutic monoclonal antibody administration. Sample matrix, tube type, clotting and centrifugation procedures, transport, storage, and freeze–thaw exposure should be standardized. Relevant collection conditions, including posture and hydration, should be controlled where feasible or documented. Intercurrent infection, changes in renal or hepatic function, and treatment modifications should be recorded as potential explanations for a changing profile rather than automatically attributed to progression of the underlying gammopathy.

Analytical continuity is equally important. Serial profiles should preferably be acquired on the same platform using a fixed preprocessing pipeline and model version. Instrument changes, reagent lots, software updates, migration alignment, and normalization procedures should be documented. When a method or platform changes, paired measurements are needed to assess comparability before trajectories are combined. Analyses based on relative signal intensities should also account for changes in total protein and other fractions, since a relative increase does not necessarily represent an absolute increase in the component of interest.

Published studies provide evidence on serial M-protein variability but do not establish thresholds for AI-derived features. Katzmann et al. estimated long-term variation in clinically stable, untreated patients with measurable monoclonal gammopathies [27]. Salamatmanesh et al. evaluated analytical and within-person biological variation and calculated reference change values using gel-based SPE in stable MGUS patients [28]. Their estimates apply to the populations, concentration ranges, methods, and sampling conditions studied; they should not be transferred directly to low-amplitude computational signatures or treated populations.

For conventional quantitative measurements, a reference change value may help assess whether a difference exceeds expected analytical and within-person biological variation, provided the estimates and statistical assumptions are appropriate [27,28]. Exceeding this threshold does not itself establish disease progression. AI-derived features require their own characterization of variability, including concentration-dependent measurement error where relevant. Percentage changes may be unstable for features near zero, and repeated evaluation of multiple features requires control of false alerts over time.

Longitudinal models should therefore account for dependence between measurements, unequal sampling intervals, and potentially informative missingness, including more frequent testing in patients whose condition is deteriorating. Baseline estimation and prediction must use only information available at the prediction time. Prospective evaluation should assess false alerts per patient over follow-up, the persistence of detected changes, and their association with independently defined clinical outcomes. Any claim of earlier detection should demonstrate additional predictive value beyond established monitoring markers and distinguish an earlier signal from an improvement in patient management.

Multimodal integration should be evaluated against a comparator that reflects the information available at the intended decision point. Depending on the application, this may include quantitative immunoglobulins, serum free light chains and their ratios, blood count parameters, renal function, calcium, and inflammatory markers. Predictor selection should follow the clinical question rather than the availability of a large laboratory dataset. A model combining these variables with SPE features may perform well without demonstrating that the electrophoretic input contributes additional information.

We propose comparing three prespecified models: a model using routinely available clinical and laboratory variables; the same model extended with conventional SPE information, including fraction measurements and relevant interpretive findings; and a further extension incorporating AI-derived SPE features. The second comparison is particularly important when the claim concerns information beyond conventional electrophoretic interpretation. Models should be developed using comparable optimization procedures and evaluated in the same independent patients against the same outcome definition. An SPE-only model may provide a secondary comparison, but cannot establish incremental value over routine assessment.

Incremental predictive performance should be assessed through changes in discrimination and, for probability-based models, calibration and overall prediction error, with confidence intervals for paired comparisons. A statistically significant association of an SPE feature with the outcome, or a high feature-importance score, does not establish useful incremental prediction. Similarly, an increase in AUC does not necessarily translate into better decisions at the thresholds relevant to practice [13,26].

The intended action should therefore be specified before evaluation. For example, a model used to select samples for immunofixation could be assessed by the reduction in confirmatory tests at a prespecified acceptable sensitivity, or by additional confirmed findings at a comparable testing workload. For prognostic applications, comparisons should use a common prediction horizon and evaluate whether changes in predicted risk would alter monitoring or management. Decision-curve analysis can estimate incremental net benefit across clinically justified thresholds [26], while prospective workflow evaluation is required to establish the consequences of acting on the predictions, as discussed in Section 5.4 and Section Recommendations for Prospective, Multicenter, and Cross-Platform Validation.

Multimodal datasets also require explicit temporal alignment and management of missing information. Only results available when the decision would be made should enter the model; confirmatory tests ordered after an AI alert must not be included as predictors of that same decision. Measurements used to define the reference outcome require particular scrutiny to avoid circular evaluation. Missingness should be examined because selective ordering of tests may encode clinical suspicion or local practice. Feature selection, imputation, and model tuning should remain within development data, with external evaluation accounting for differences in test availability and ordering pathways [13,14].

Integration with proteomic, metabolomic, or genomic data may help investigate the biological origin of candidate SPE features. Such integration addresses a different question from whether routinely available data support better clinical decisions. Claims of clinical value should therefore specify the contribution attributable to SPE, the comparator used, and whether the evidence concerns predictive performance, estimated decision benefit, or observed effects on care.

### Recommendations for Prospective, Multicenter, and Cross-Platform Validation

We propose a staged evaluation comprising prospective assessment of model performance, investigation of transportability across centers and platforms, and evaluation of the AI-assisted workflow. Before recruitment, the protocol should specify the intended population, clinical task, model output, reference assessment, primary endpoint, and acceptance criteria. The model version, preprocessing pipeline, and decision thresholds should be fixed before evaluation. Any local adaptation should use separate data, with performance before and after adaptation reported separately [13,14].

Prospective performance assessment should initially run alongside routine testing, with AI outputs withheld from clinical decision-making. Consecutive eligible patients should be enrolled at participating centers, including sites that contributed no data to model development. Eligibility should reflect the intended use, distinguishing initial investigation from monitoring of established disease. For evaluation in previously unseen patients, development and evaluation cohorts should not overlap at the patient level. Serial specimens should be identified and analysed as dependent observations. This phase can assess diagnostic performance and estimate potential review volumes, but cannot establish the effects of AI on clinical decisions.

The reference assessment should be appropriate to the target and interpreted without access to AI results. For M-protein detection, a prespecified confirmatory-testing strategy should include AI-positive and AI-negative samples, rather than verifying only flagged profiles. If complete verification is infeasible, a predefined sampling strategy and an analysis addressing partial verification are required. Discordant and indeterminate findings should undergo documented adjudication without assuming that either the algorithm or routine interpretation is correct. Claims concerning novel systemic signatures require independently defined clinical outcomes or phenotypes rather than conventional electrophoretic labels alone.

Cross-platform evaluation should separate analytical effects from differences in patient populations. We propose combining routine cohorts from each center with a shared panel of patient-derived specimens analysed on the platforms under evaluation. Where feasible, more than one center should use each platform, reducing confounding between site and instrument. Aliquot preparation, transport, storage, and analysis timing should be controlled and documented. The shared panel should include low-concentration and beta-migrating components, relevant interferents, and representative negative profiles. Such a panel tests analytical comparability; its enriched composition does not provide routine-population predictive values. Models requiring platform-specific preprocessing should be evaluated using that predefined configuration, without assuming that numerical signals are interchangeable across systems.

Performance should be reported overall and by center, platform, and prespecified clinical subgroup, with confidence intervals and methods accounting for clustering and repeated measurements. Diagnostic classification, probability calibration, and quantitative agreement require different endpoints. Sample size should be justified by the precision required for the primary endpoint and the expected number of informative positive and negative cases, allowing for subgroup analyses, clustering, and incomplete reference assessment. A large pooled cohort should not substitute for adequate evaluation within the settings covered by the intended claim [13,14].

After predefined performance requirements are met, a prospective comparative study should evaluate the AI-assisted pathway against usual practice using the benefit, safety, and workload endpoints described in Section 5.4. Randomization at the patient or cluster level may be appropriate, depending on the risk of contamination between workflows; nonrandomized designs should address temporal changes and differences between sites. Initial monitoring should include predefined criteria for investigating performance deterioration or suspending automated actions. This final phase tests whether the validated model improves the intended workflow, rather than merely reproducing retrospective classification results.

## 7. Conclusions

AI is reshaping the interpretation of serum protein electrophoresis, but the maturity of its clinical applications remains uneven. The strongest evidence concerns automated recognition of established abnormalities, particularly monoclonal proteins, for which ML and DL models have achieved high diagnostic performance in large retrospective datasets and, in selected studies, independent validation [5,6,7,8,9,10,11,12]. These findings establish the value of AI for improving the consistency, scalability, and potentially sensitivity of conventional SPE interpretation. However, they predominantly demonstrate better recognition of known diagnostic targets rather than discovery of new clinically meaningful electrophoretic information.

This distinction defines an important gap in the current literature. While previous research has largely focused on algorithm development and performance for predefined SPE abnormalities, considerably less attention has been given to what should happen after an AI model detects information beyond conventional visual interpretation. Modern electrophoretic platforms provide machine-readable numerical signals that may expose low-amplitude, distributed, or longitudinal features not explicitly considered during routine interpretation. Yet the biological meaning and clinical relevance of these additional features remain largely unresolved. A computationally detectable signal may represent early disease-related information, but it may equally reflect physiological variability, analytical effects, or platform-specific characteristics.

This review therefore shifts the focus from algorithmic performance to signal validation. We propose evaluating additional AI-detected electrophoretic features across four complementary domains: analytical validity, biological validity, clinical validity, and clinical utility. The evidence required depends on the intended claim and clinical use; computational detectability or reproducibility alone does not establish clinical value.

This framework also provides a basis for distinguishing established applications, such as M-protein recognition, from emerging or conceptual uses in which biological attribution and clinical relevance have yet to be demonstrated.

The next phase of AI-assisted SPE research should therefore be driven less by increasing model complexity than by determining whether computationally accessible information provides incremental value beyond existing laboratory and clinical pathways. AI should be viewed neither simply as an automation layer nor as a replacement for expert interpretation, but as a tool for determining whether underused electrophoretic signal features can be transformed into analytically reproducible, biologically meaningful, and clinically actionable laboratory information.

Ultimately, the future value of AI in SPE will depend not on how much additional signal algorithms can detect, but on whether that signal can be shown to matter for laboratory diagnosis and patient care.

## Figures and Tables

**Table 1:** Datasets, validation strategies, and limitations of representative computational approaches to SPE interpretation.

Study	Approach and Dataset	Validation and Reference Assessment	Main Contribution and Limitations
**Ognibene et al.** [15]	CASPER neural-network algorithm; 59,516 samples from six Italian laboratories.	Separate training, validation, and evaluation groups; multicenter assessment of electrophoretic profile recognition.	Early multicenter evaluation of computer-assisted interpretation. Sensitivity was 93.0%, but specificity was 47.4%, with variation between centers. The results supported an adjunctive role rather than replacement of visual inspection.
**Chabrun et al. [5]**	SPECTR deep-learning system for quantitative and qualitative SPE interpretation; 159,969 profiles in the development dataset.	Internal evaluation and an independent external cohort of 70,362 profiles; expert-annotated interpretations and an additional comparison with nine experts.	Large-scale external evaluation across several interpretation tasks. Performance against expert-defined categories should be distinguished from detection against an independent biochemical reference. Overall normal-versus-abnormal classification metrics are not specific to M-protein detection.
**Sopasakis et al.** [7]	Comparison of 26 decision-tree methods using numerical capillary electrophoresis data; 67,073 samples from 32,490 patients.	Internal training/test evaluation. Labels were based on expert assessment of capillary and gel electrophoresis, with immunofixation performed for suspected M-proteins.	Demonstrated the feasibility of tree-based analysis of numerical profiles. External transportability was not established. Repeated samples and selective confirmatory testing require consideration when interpreting independence and reference-label accuracy. Rare isotypes were insufficiently represented for the isotype-classification task.
**Elfert et al.** [8]	Four feature-based algorithms and one CNN; 69,722 individuals, including 4273 immunotyping-positive and 65,449 negative samples.	Development used 68,722 profiles; a held-out set of 1000 samples was also assessed by ten experts. Immunotyping provided the reference labels.	A common dataset enabled direct comparison of model types and experts. Random forest achieved high specificity but lower sensitivity than the experts. The internal test design does not establish performance in other laboratories or populations.
**Lahtiharju et al.** [9]	Locally trained neural networks based on the SPECTR methodology for fractionation, classification, and paraprotein integration; first samples from 34,625 patients.	Training and validation groups comprised 25,969 and 8656 patients, respectively. Immunofixation results were available for a subset of the cohort.	Evaluated the methodology in a Finnish clinical setting while avoiding repeated patient samples. Local model training should be distinguished from external testing of an unchanged pretrained model. Incomplete immunofixation coverage limits uniform reference verification.
**Shen et al.** [10]	AIRSPE image-based CNN system; 166,003 profiles for training and optimization.	A subsequent same-center test set of 10,014 samples and a second-center test set of 1861 samples; both required available immunofixation results.	Assessed M-spike detection against immunofixation, including low-concentration components. Higher internal-test sensitivity than manual interpretation was accompanied by lower specificity. Selection of samples with immunofixation results limits extrapolation to unselected routine requests.
**Wang et al.** [11]	Comparison of ML and DL models and input representations; 85,026 SPEP results for development and validation, with a separate test dataset of 1079 samples.	Additional testing of selected models on a separate dataset of 1079 samples; localization compared with three clinical experts.	Evaluated classification and localization, with favorable results for a U-Net–Transformer model. Diagnostic classification and localization performance do not establish improvement in clinical decisions or routine workflow.
**Cheung et al.** [12]	Three neural networks for fractionation, classification, and localization/quantification. Source datasets comprised 41,448 profiles from Princess Margaret Hospital and 24,501 from Tuen Mun Hospital.	Models were developed using the first hospital’s data and evaluated on 24,238 profiles from the second hospital. Reference interpretations were provided by one or two pathologists.	Provided a cross-hospital retrospective evaluation using the Sebia Minicap system. Agreement with reported interpretations does not establish independent biochemical confirmation. Correlation of peak quantities does not by itself demonstrate quantitative agreement or interchangeability.

*Abbreviations:* CNN, convolutional neural network; DL, deep learning; ML, machine learning; SPE/SPEP, serum protein electrophoresis. *Note:* Dataset sizes refer to profiles, samples, or individuals as reported and should not be treated as equivalent denominators. Study-specific outcomes and reference assessments differ; the table does not support a numerical ranking of algorithms across studies. Evaluation in another center, evaluation on another platform, and evaluation after local retraining provide different evidence about transportability.

**Table 2:** Proposed operational criteria for validating additional AI-detected SPE features.

Validation Domain	Operational Question	Assessment Strategy and Endpoints	Criterion Supporting the Intended Claim
**Analytical validity**	Can the feature and model output be measured reproducibly under the conditions in which they will be used?	Analyse replicate aliquots within and between runs and assess relevant effects of sample storage, reagent lots, instruments, and preprocessing. Fix the feature definition, preprocessing pipeline, and model version before evaluation. Assess feature variability, prediction agreement, and changes in classification near decision thresholds. Evaluate additional sites and platforms when these are included in the intended use.	Measurement variability and prediction instability remain within prespecified limits appropriate to the intended decision. Any restriction to particular platforms or sample conditions is documented. Reproducibility of the prediction alone does not establish reproducibility or biological meaning of the underlying feature.
**Biological validity**	What evidence supports the proposed biological interpretation of the feature?	Compare the feature with independent measurements selected for the biological claim, such as immunotyping, mass spectrometry, or targeted protein measurements. Where appropriate, use controlled addition, depletion, or interference experiments. Assess alternative explanations, including inflammation, renal or hepatic dysfunction, and treatment exposure.	Independent findings support the proposed attribution, with relevant alternative explanations examined. Association with a diagnostic label alone is insufficient to establish molecular identity or disease specificity. If attribution remains unresolved, the feature is described as a computational signature of uncertain biological origin.
**Clinical validity**	Does the feature or model identify or predict the specified clinical target in the intended population?	Evaluate a fixed model in an independent, representative cohort using a reference standard or outcome definition appropriate to the target. Report sensitivity, specificity, and predictive values at prespecified thresholds for diagnostic applications; assess discrimination and, for probabilistic predictions, calibration. Include confidence intervals and relevant subgroup analyses. For prognostic claims, specify the prediction horizon and establish that measurements precede the outcome.	Performance meets prespecified, clinically justified requirements in the target population, with uncertainty reported. Claims of added information require comparison with existing clinical and laboratory predictors; claims of earlier detection require longitudinal evidence.
**Clinical utility**	Does using the AI output improve the intended laboratory or clinical pathway compared with current practice?	Prospectively compare AI-assisted and usual workflows. Prespecify the action triggered by the output and measure appropriate confirmatory testing, missed clinically relevant abnormalities, unnecessary investigations, turnaround time, and review workload. Assess patient outcomes when these form part of the intended claim.	The AI-assisted pathway demonstrates a prespecified benefit without an unacceptable increase in missed findings, unnecessary investigations, or workload. The conclusion is restricted to the outcomes evaluated: improved laboratory efficiency does not by itself establish improved patient outcomes.

*Note:* Assessments should be proportionate to the intended use. A model restricted to one platform does not require demonstrated compatibility with all electrophoretic systems, but its validation does not support use on untested platforms. Analytical reproducibility, biological attribution, clinical performance, and effects on care support different claims and should be reported separately.

## Data Availability

No new data were generated in this study. All information discussed in this review was derived from previously published studies, which are appropriately cited in the manuscript.

## Abbreviations

The following abbreviations are used in this manuscript:

**AI**	Artificial intelligence
**AIRSPE**	Artificial Intelligence-based Recognition of Serum Protein Electrophoresis
**AUROC**	Area under the receiver operating characteristic curve
**CNN**	Convolutional neural network
**DL**	Deep learning
**1D-CNN**	One-dimensional convolutional neural network
**ML**	Machine learning
**M-protein**	Monoclonal protein
**PROBAST + AI**	Prediction Model Risk of Bias Assessment Tool plus Artificial Intelligence
**SPE**	Serum protein electrophoresis
**t-mAb**	Therapeutic monoclonal antibody
**TRIPOD + AI**	Transparent Reporting of a Multivariable Prediction Model for Individual Prognosis or Diagnosis plus Artificial Intelligence
